# Effects of climate change on parasites and disease in estuarine and nearshore environments

**DOI:** 10.1371/journal.pbio.3000743

**Published:** 2020-11-24

**Authors:** James E. Byers

**Affiliations:** Odum School of Ecology, University of Georgia, Athens, Georgia, United States of America; University of York, UNITED KINGDOM

## Abstract

Information on parasites and disease in marine ecosystems lags behind terrestrial systems, increasing the challenge of predicting responses of marine host–parasite systems to climate change. However, here I examine several generalizable aspects and research priorities. First, I advocate that quantification and comparison of host and parasite thermal performance curves is a smart approach to improve predictions of temperature effects on disease. Marine invertebrate species are ectothermic and should be highly conducive to this approach given their generally short generation times. Second, in marine systems, shallow subtidal and intertidal areas will experience the biggest temperature swings and thus likely see the most changes to host–parasite dynamics. Third, for some responses like parasite intensity, as long as the lethal limit of the parasite is not crossed, on average, there may be a biological basis to expect temperature-dependent intensification of impacts on hosts. Fourth, because secondary mortality effects and indirect effects of parasites can be very important, we need to study temperature effects on host–parasite dynamics in a community context to truly know their bottom line effects. This includes examining climate-influenced effects of parasites on ecosystem engineers given their pivotal role in communities. Finally, other global change factors, especially hypoxia, salinity, and ocean acidity, covary with temperature change and need to be considered and evaluated when possible for their contributing effects on host–parasite systems. Climate change–disease interactions in nearshore marine environments are complex; however, generalities are possible and continued research, especially in the areas outlined here, will improve our understanding.

## Abstract 2

Information on parasites and disease in marine ecosystems lags behind terrestrial systems, increasing the challenge of predicting responses of marine host-parasite systems to climate change. This Essay highlights five general principles to guide the study of the response of marine host-parasite interactions to climate change, including the effects of temperature, oxygen, acidity, and salinity.

## Introduction

Parasites and other infectious agents are common in marine ecosystems. They are important to understand both because they are natural components of ecological systems and because of their ability to cause outbreaks and disease-induced mortality. Parasites and disease, like all other biological entities, are temperature sensitive and all perform optimally within an evolved temperature range. This statement seems obvious, but it belies early thinking in disease ecology, which hypothesized that a warmer world would equal a sicker world [1]. This hypothesis initially gained support because extreme examples are more easily noticed and because of a publication bias where largely only positive results are published. With more scrutiny, we now know that this hypothesis is too simplistic and does not consistently hold [2–6].

In the marine realm, we should be especially cautious about being drawn prematurely to this hypothesis (or any other overly general hypothesis) because we know that studies of marine host–parasite pairs are taxonomically biased and woefully undersampled to conclude reliable ecological patterns. For example, Lafferty [7] tabulated only 102 “notable marine diseases” in the ecological literature across 8 disparate host taxa, from plants to mammals. For comparison, in terrestrial systems, Wiethoelter and colleagues [8] found 118 infectious diseases reported in the literature looking just at the wildlife–livestock interface. Host–parasite interactions may differ between marine and terrestrial systems because environmental and organismal differences between these 2 biomes are large. For instance, compared to terrestrial species, marine organisms have different life histories, including a preponderance of open population structure, which makes environmental transport as a disease transmission mode likely far more common, including over long distances [9]. Here, I propose 5 generalizable aspects of marine parasites and disease. Our understanding of each of these aspects continues to sharpen, thus providing exciting, fruitful avenues for continued research.

### Thermal performance curves to enable prediction

Instead of drawing conclusions across the relatively small number of marine diseases that have been studied in an ecological context, a more productive approach to understanding temperature’s mechanistic effects may be to instead examine changes in host–parasite interactions of individual species, including across the entire life spans of host and parasite. In this regard, the comparison and matching of thermal performance curves of host and parasite have helped sharpen our thinking on how to predict temperature effects [10]. For this approach, one needs to quantify temperature effects on host and parasite vital rates and parasite transmission rates. By comparing these curves for host and parasite, the effect of temperature to differentially benefit either the parasite or the host can be obvious. However, these data are often tedious to generate. Their use has largely been in the realm of insects because of their short generation times (which facilitate easier vital rate quantification), ectothermy, and their importance as disease vectors [11]. Many marine species, especially invertebrates, have similar characteristics and would thus be good candidates for thermal performance curve construction.

**Fig 1:**
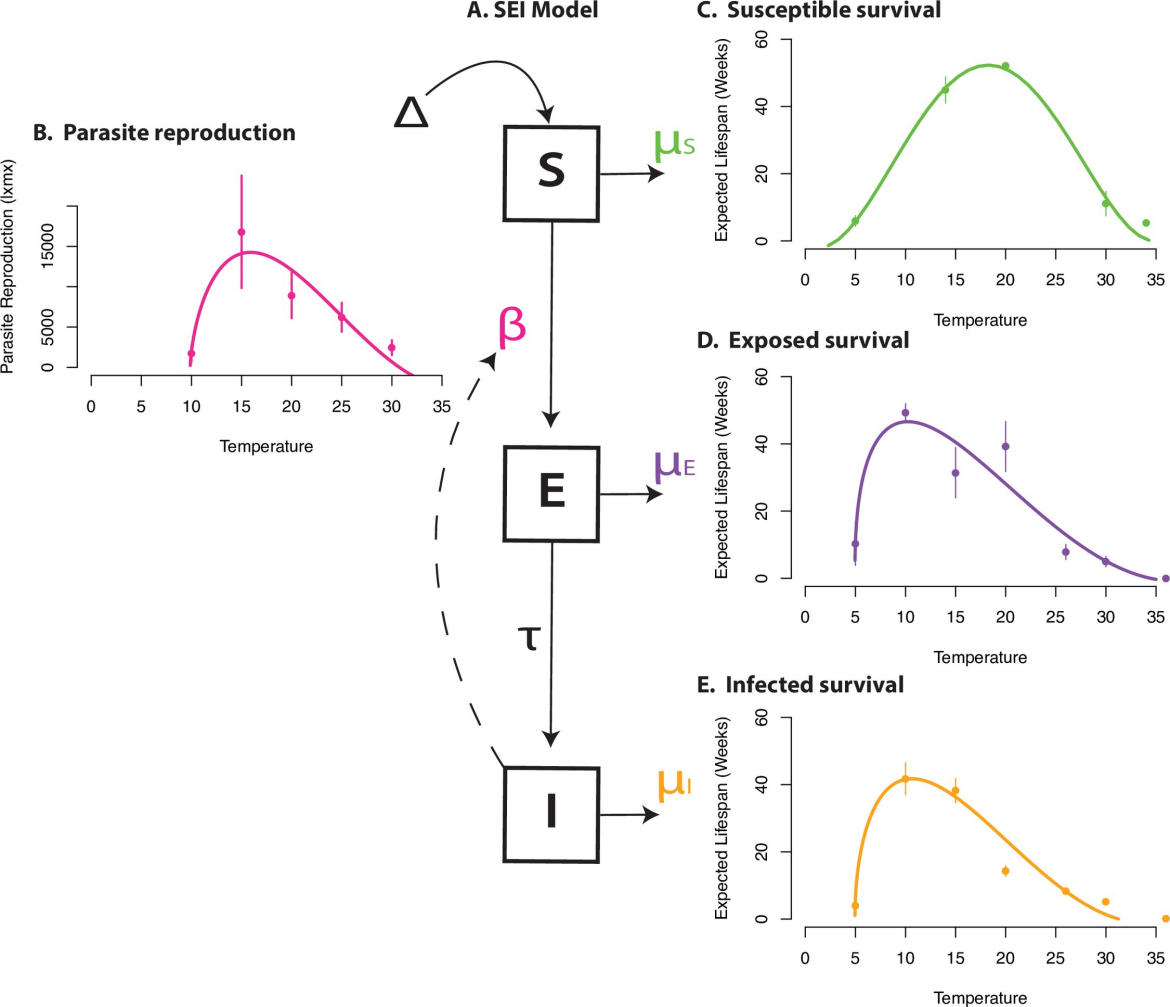
(A) An SEI (susceptible, exposed, infective) model schematic for transmission of the parasitic rhizocephalan, *Loxothylacus panopaei*, in the mud crab, *Eurypanopeus depressus*. Susceptible hosts, *S*, are recruited at a constant weekly rate, Δ, during the recruitment period and die at per capita rate μ_s_ (= 1/expected life span). Susceptible hosts become parasitized at per capita rate *βSI*, where the transmission rate, *β*, is an agglomerate parameter assumed proportional to larval parasite production, and *I* is the number of infectious hosts with the reproductively mature parasite stage. Exposed hosts, *E*, are infected with reproductively immature parasites that develop at rate *τ*. Exposed and infectious hosts have respective mortality rates μ_E_ and μ_I_. Model parameters labeled in color are temperature dependent and are parameterized based on thermal performance curves fit to experimental measurements of (B) parasite reproduction (lifetime reproduction, l_x_m_x_) and host survival for each infection status: (C) susceptible, (D) exposed, and (E) infected. Figure reproduced from [14].

Although thermal performance curve construction is a valuable start, often, a comprehensive model may be needed to compile the various temperature-dependent responses to predict the net effects on the host and parasite dynamic. This is because temperature affects many rates of host and parasite simultaneously, perhaps in opposing directions [12]. For example, temperature stress may increase host susceptibility, increasing transmission. But increased temperatures may also increase infected host mortality, decreasing transmission [13]. Gehman and colleagues [14] is 1 of the only marine examples constructing and comparing host–parasite thermal performance curves to model population-level effects, and it was conducted on a short-lived marine crab *Eurypanopeus depressus* and its rhizocephalan barnacle parasite, *Loxothylacus panopaei* (Fig 1). Interestingly, Gehman and colleagues [14] predicted that warming temperatures would be more detrimental for the parasite than the host crab. Shields [15] compiled thermal performance curves for 3 other crustacean host–parasite pairs and found the parasite likely to benefit differentially from warmer temperatures. More examples like these for marine host–parasite pairs could enable better prediction and broader generalities.

### Shallow and intertidal areas more heavily affected by temperature change

Although thermal performance curves may be a gold standard for predicting host–parasite dynamics in a changing climate, they are labor intensive to quantify. Thus, it may be useful to seek some generalities to guide our expectations. For example, with the exception of marine mammals, seabirds, and a few fish species, marine animal species are ectotherms and are therefore highly affected by environmental temperature. Estuarine environments should be some of the most susceptible marine habitats to temperature swings. Water temperature is most affected in shallow areas where contact with the air and solar energy is greatest, and thus temperature variability is the highest. Furthermore, because air is a poorer temperature buffer than water (due to its low-specific heat relative to water), this suggests that temperature variability and extremes will affect intertidal organisms more than subtidal ones. In fact, some of the strongest temperature gradients are vertically up the intertidal zone where organisms are increasingly exposed to air. Gradients of parasitism in species spanning the subtidal to the high intertidal zone are often evident even across small spatial scales of only a few meters [16–18].

However, often, these are observational data and not experimental, so it is not known if temperature alone is the causal factor driving the patterns. For example, differential emersion from water along a vertical intertidal gradient changes exposure to oxygen, food, predators, and vectors, which can also influence parasite patterns [19]. However, Malek and Byers [20] provide a sound example with intertidal oysters *Crassostrea virginica* in the southeastern United States that are infected by a lethal protozoan parasite, *Perkinsus marinus*. Intertidal (air exposed) oysters have higher *P*. *marinus* infection intensity and marginally higher infection prevalence than subtidal (submerged) oysters. In a lab experiment, Malek and Byers [20] exposed oysters daily to heated air temperature treatments and found that parasite infection intensity peaked at 35°C compared to oysters exposed at lower temperatures and controls held subtidally the whole time. Thus, during summer, air temperatures, which are much warmer than water, can causally increase infection intensity of intertidal oysters. Also, Ben-Horin and colleagues [21] experimentally examined withering syndrome in intertidal black abalone (*Haliotis cracherodii*) and found that heightened temperature variability, as opposed to higher mean temperature alone, increased the susceptibility of black abalone to infection.

It is important to recognize that not all parasitized hosts are affected by temperature. Studer and colleagues [22] examined trematode metacercariae abundance in cockles along a latitudinal and sea temperature gradient along the New Zealand coastline and found no relationship. Especially in observational, large-scale studies, where latitude is used as a proxy for temperature, the temperature sensitivity of host–parasite systems may be offset by locally important factors, including local adaptation, which confer resilience against climate change.

In sum, intertidal and shallow water marine habitats are likely to be areas of highest temperature effects as the climate warms. The heightened exposure of organisms to increased mean temperature and variability in these habitats, coupled with the proximity and relative ease of sampling these nearshore environments, makes them useful sentinels of the effects of climate change on disease dynamics in marine systems.

### Temperature-dependent intensification of impacts on hosts

For those parasites for which increases in temperature do not cross their lethal limits, we might expect their intensity and pathology in infected host individuals generally to increase. Intensity and pathology are often positively temperature dependent. This positive association with temperature occurs for 2 primary reasons. First, temperature increases parasite metabolism, which increases its feeding, and often its replication inside a host, accentuating damage [23,24]. Second, the host experiences more environmental stress, deteriorating its resistance to the infecting pathogen. For ectotherms, warmer temperatures boost organismal metabolism, while simultaneously driving down oxygen saturation in the water. Low oxygen is a particularly influential environmental stressor, especially during summer in shallow water environments and particularly if they are eutrophic [25]. The stress of low oxygen associated with increased temperature can weaken hosts and exacerbate disease impacts [26]. In these cases, it is the interaction of the parasite with the temperature-dependent stressor causing impact. To illustrate these concepts further, below are 3 examples of temperature-dependent pathology of diseases in 3 disparate estuarine or nearshore organisms.

Neoplasia is a diffuse tumor of the hemic system that infects many bivalve mollusks, including the soft-shell clam *Mya arenaria*. Although, like most cancers, it is initiated by genetic mutation, the neoplasia can be horizontally transmitted between individuals [27]. Böttger and colleagues [28] documented the highest prevalence of *M*. *arenaria* with terminal neoplasia from an estuary in Massachusetts in December (9.5%) when seawater temperatures were low and the lowest prevalence in July (1.1%) when seawater temperatures were highest. These results may indicate vulnerability of neoplastic clams to seasonal increases in environmental temperature and resulting oxidative stress [28]. If correct, it suggests that if temperatures stay cold, the clams can harbor infection longer and stay alive, presumably because the metabolisms of both the host and the neoplasia are diminished.

*Hyalophysa lynii* is a newly identified apostome ciliate parasitizing penaeid shrimp in the southeastern US [29]. The ciliate lives in the gills of the shrimp, initiating an immune response by the host that results in melanized nodules in shrimp gill tissue, resulting in the common name of the disease as shrimp black gill. These nodules interfere with O_2_ exchange and reduce physical endurance and escape responses compared to shrimp without nodules [30]. As oxygen concentrations drop with increasing temperature, the physical impairment intensifies stress on the host. Because temperature stress seemingly accentuates disease pathology, it is not surprising that the highest prevalence of the late-stage symptomatic expression of the disease is found in late summer as water temperatures peak [31].

Finally, sea star wasting disease (SSWD) affects many species of sea stars, particularly in the northeastern Pacific. Although uncertainties exist about the agent of disease and triggers of its outbreaks, one agreed-on fact is the disease’s diminished effects in cooler water (e.g., [32]). With a laboratory experiment, Kohl and colleagues [33] tested whether cool temperatures, representative of average winter temperatures in Washington, could slow the progression of morbidity and even prevent SSWD mortality in *Pisaster ochraceus* compared to average summer temperatures. Although the cooler temperatures did not prevent SSWD mortality, sea stars in cooler water lived more than twice as long as stars held at summer temperatures. These data are consistent with experimental studies and field observations during SSWD outbreaks that seem to occur during, or immediately following, temperature increases [34–36] and support that changes in coastal water temperatures have influenced 1 of the most recent, ecologically impactful marine diseases [33].

### Host–parasite dynamics should be studied in a community context

Temperature is likely to affect not only the direct effects of parasites on their hosts but also the parasites’ secondary effects. These secondary effects involve interactions with other species in the community that generate indirect effects, intensifying effects on the host, or affecting the larger ecological community. Effects of parasites on communities may be especially large if the parasites infect prominent consumers or habitat-forming species and ecosystem engineers because of these species’ fundamental role in community structure [37,38]. For example, seagrass beds, reef-building coral, and oysters are ecosystem engineering hosts that are increasingly succumbing to disease as temperature rises and whose decreases will likely cascade to affect the communities for which they play a critical role provisioning habitat [39,40]. In a well-documented example, high water temperature (26°С) in the Wadden Sea in 1990 stimulated a spike in trematode cercariae production in infected snails (*Hydrobia ulvae*), which resulted in metacercarial hyperinfection of the second intermediate amphipod host (*Corophium volutator*) and its consequent mass mortality [41,42]. The population crash of *C*. *volutator* strongly affected the ecosystem because the normally abundant amphipods are ecosystem engineers that build tubes from sand that stabilize the benthic sediment. The absence of the amphipods resulted in substrate erosion and alterations of productivity and benthic community structure, including the loss of several macrofaunal groups [43,44].

Because they involve indirect effects, secondary effects of parasites on their hosts and communities cannot be studied in isolation, but rather need to be embedded in a community context. We need to simultaneously understand how the influence of this community context on host–parasite dynamics might change with increasing temperatures. For instance, Cohen and colleagues [45] modeled how rising temperatures will affect polar sea ice extent, in turn influencing marine mammal distributions. They suggest that increased abundance and aggregations of seals will lead to subsequent increases in the pinniped disease brucellosis, with a high possibility of spillover of this generalist bacteria disease to other hosts.

Two further detailed examples illustrate this concept of secondary effects that is simple in theory, but whose influence is likely highly underestimated, and requires much greater effort to quantify. In a multispecies community context, a disease can have major pathological effects without directly killing its host. As mentioned above, black gill disease compromises respiration of the infected shrimp host. When resting, oxygen demand for the infected shrimp is low enough that it can readily survive. However, increasing temperatures decrease oxygen levels, making the already hard job of oxygen extraction even harder. Furthermore, when infected shrimp are made to exert themselves in simulated predation trials, they tire more quickly and exhibit fewer escape behaviors [30]. Gooding and colleagues [46] took this a step further to embed these isolated, individual physiological responses to disease into a real-world, multispecies context. They exposed symptomatic black gill and asymptomatic shrimp to 3 different fish and crab predators in a prey choice mesocosm experiment at warm water temperatures. The infected symptomatic shrimp were differentially preferred by the predators 1.4 to 3 times more than asymptomatic shrimp [46]. This finding underscores the dramatic influence that community-level interactions can have on a host–parasite interaction. In fact, 1 of the largest effects of the parasite may be its enhancement of the host’s vulnerability to predators—an effect that would not be seen unless the host–parasite relationship is examined in a multispecies context, which is seldom done.

**Fig 2:**
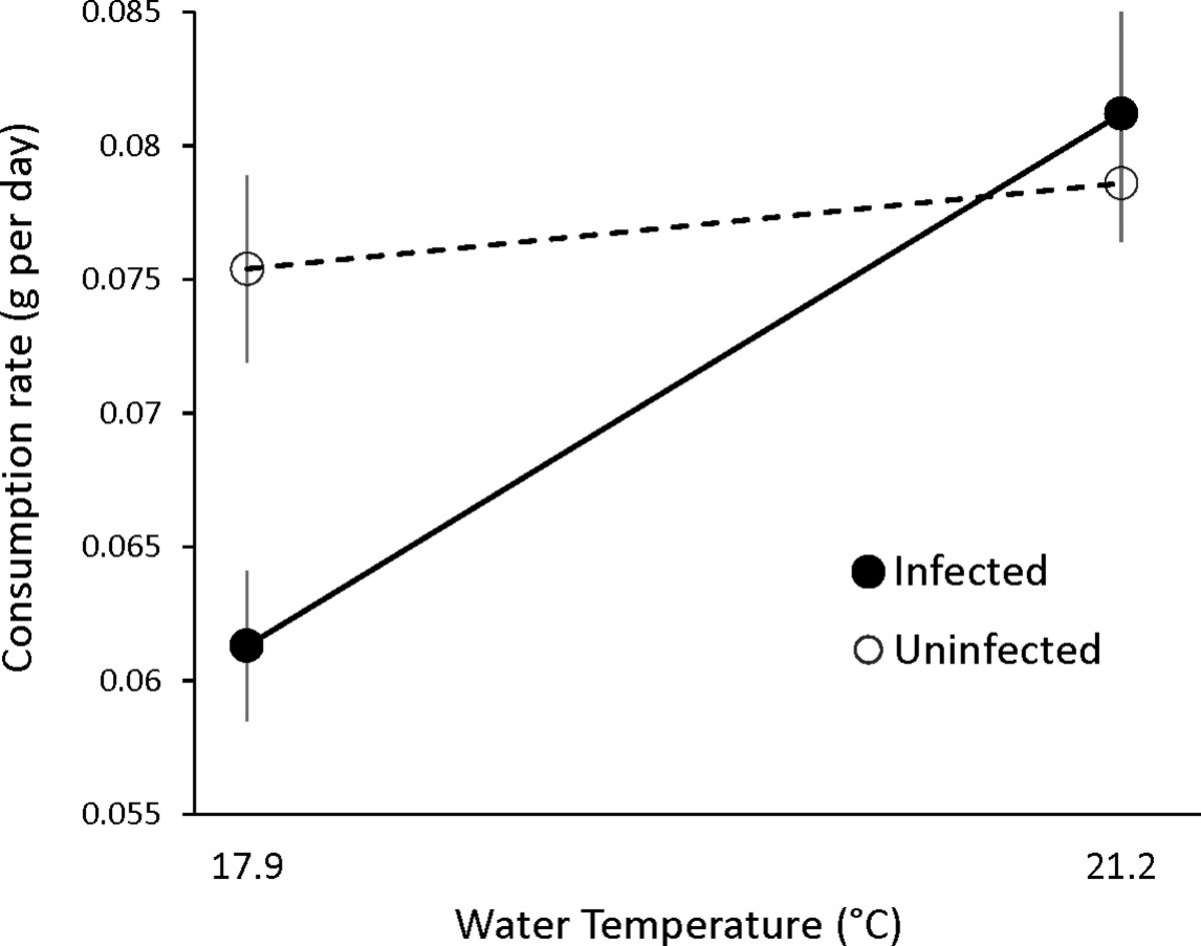
Consumption rate of the green alga *Ulva lactuca* by large uninfected *Littorina littorea* snails (open circles, dashed line) and trematode-infected snails (darkened circles, solid line) at water temperatures of 17.9°C and 21.2°C. Values are means ± SE. Figure reproduced with permission from [48], Inter-Research 2009. SE, standard error.

The shrimp black gill example illustrates a parasite increasing indirect effects on the host at higher temperatures. A second example, similar to the parasite-induced crash of *C*. *volutator* in the Wadden Sea, illustrates a parasite’s temperature-dependent indirect effects on the larger ecological community in which it is embedded. Wood and colleagues [47] showed that the intertidal snail *Littorina littorea*, which is the dominant herbivore in the system, ate 30% less algae when parasitized with a trematode, *Cryptocotyle lingua*. The authors then experimentally quantified that as a consequence of their altered grazing, in just 1 month, populations of infected snails in the field had created different algal communities compared to uninfected populations. Following with the same host species, Larsen and Mouritsen [48] experimentally demonstrated that temperature interacted with trematode infection to affect the rate of algal consumption by the snail host. Specifically, although parasite infection decreased snail grazing rate, temperature worked to increase it, not only by increasing the snail’s metabolism but also by increasing the energy demands placed on the host by the parasite. Thus, temperature positively affected consumption by snails, but particularly so for trematode-infected hosts. An increase in temperature of just 3°C was enough to neutralize the negative impact trematodes otherwise have on snail consumption (Fig 2).

### Factors that covary with temperature change also affect host–parasite systems

With global change, increases in temperature do not occur in a vacuum. Several factors that influence disease prevalence and intensity covary with temperature. This can make elucidating the causal effect of temperature on parasitism tricky; in some cases, temperature may even interact with these co-variables to create synergistic effects. Dissolved oxygen is one such variable that was discussed above. These co-variables also include likely influential anthropogenic factors on host–parasite dynamics such as human fishing pressure, pollution, and impervious surface area [49] that are increasing over time as temperature also rises. Introductions of non-native species also continue to escalate, bringing novel hosts and parasites in contact with one another [50–52]. Another prominent factor increasing concomitantly with global temperature in coastal environments is the variability in precipitation and salinity.

Global climate change is increasing variability in precipitation patterns [53]. The local balance of precipitation and evaporation determines sea surface salinity; thus, salinity varies spatially, with higher salinities found in the evaporation-dominated midlatitudes. This balance can be sensitive, especially nearshore where water is shallow and thus atmospheric inputs to the ocean (including runoff from land) represent a larger proportion of the water volume. The parasitic dinoflagellate *Hematodinium* sp. parasitizes blue crabs (*Callinectes sapidus*) throughout estuaries of the Atlantic coast of the US. Infections in blue crabs have only been reported from areas where salinity is >11 [54]. Experimental exposure of infective stages (dinospores) from in vitro cultures at salinities <15 quickly inactivated them [54]. Thus, at low salinities, the parasite appears incapable of transmission, which explains the lack of natural infections in blue crabs at low salinities. Large catches of blue crab are positively correlated with high river flow, suggesting that the availability of fresh water is normally beneficial to the health of the crab population [55]. In fact, blue crabs are known to migrate up-estuary into freshwater, perhaps because this serves as a refuge from parasites like *Hematodinium* that require high salinity.

In 2002, the blue crab population in Georgia and the associated fishery crashed [56]. In 2003, landings were depressed 80% compared to historic averages [57]. An outbreak of *Hematodinium* sp. stimulated by increased salinities was thought to be a causal factor in the declines. In the early 2000s, extensive droughts throughout much of the southeastern US increased temperatures and reduced stream inflow and precipitation in estuaries, greatly increasing their salinity. For example, in 2002 in Georgia, the drought increased the average salinity in several estuaries to high levels (>30) [56]. Thus, the blue crab decline was postulated to be due to severe regional drought that increased coastal salinities, removing freshwater refuges for blue crabs and allowing *Hematodinium* to overwhelm the host population [57,58].

Another important climate change variable is atmospheric CO_2_, which is rapidly being absorbed by the ocean and expected to decrease the pH of the ocean by 0.3 to 0.5 pH units by 2100 [59,60]. With the exception of roughly 2 dozen publications, the effects of ocean acidification (OA) on parasitism have largely been neglected. Parasites exposed to OA are challenged by similar problems as all organisms, namely decreased abilities to regulate ions and conduct calcification. The few experimental studies that have been done have largely focused on trematodes and shown that generally, exposure to high CO_2_ seawater reduces the survival of free-living trematode cercariae and external metacercarial cysts [61–63]. But OA’s largest effect is stressing hosts, which alters their susceptibility to parasites [64]. For example, Harland and colleagues [65] showed experimentally that despite high mortality of the transmitting cercarial stage of a trematode under low pH conditions [62], the second intermediate amphipod hosts were more susceptible at low pH, and this factor outweighed the reduced life span of infective cercariae, resulting in more amphipod infections at low pH. Other studies also show this same pattern where stress to the host from low pH seems to outweigh stress to the parasite (e.g., [63,66,67]).

## Conclusions

Predicting the influences of climate change on host–parasite dynamics is an increasingly important goal. Focusing future research priorities on the areas outlined above can help achieve that goal. For example, we are improving our approach on how to predict effects of temperature on disease. Quantification and comparison of host and parasite thermal performance curves is a smart (but labor intensive) approach. However, there are also some other areas in which we can focus. With the exception of coral reefs, most of our information on parasites and disease comes from temperate regions of developed nations. The tropics and poles are especially important to study because insight into future temperature effects may come from better understanding host–parasite dynamics across existing broad temperature gradients that naturally occur with latitude. Also, studies that quantify site-based, long-term temporal variability in host–parasite dynamics (e.g., [68,69]) can be examined for correlations between parasites and naturally varying climatic factors. Finally, trematode parasites and snail hosts are overrepresented in the literature, as are hosts that are commercially exploited or farmed. Achieving taxonomic balance in studies will ensure that our understanding of climate’s influence is representative.

As these more detailed data are gathered, there may be some generalities to guide our expectations. In marine systems, shallow, and especially intertidal, areas will experience the biggest temperature swings and thus likely see the most changes to ectothermic host–parasite dynamics. Temperature-dependent intensification of impacts will not always occur, but for some responses like parasite intensity or host pathology, there may be a biological basis to expect intensification more often than not. Because secondary mortality effects and indirect effects of parasites can be important, we need to study temperature effects on host–parasite dynamics in a community context to better understand their net effects. Focusing on diseases of foundation species, keystone species, and ecosystem engineers, like coral, oysters, seagrass, and sea stars, seems especially warranted given the large potential for disease influences on the host to amplify to affect whole communities. Finally, other global change factors that covary with temperature change need to be considered and evaluated for their contributing, and possibly offsetting, roles. In this vein, more experimental work would help, especially multifactor experiments that can address interactive effects of different climate variables (e.g., [70]). As in any system, the effects of climate change–disease interactions in nearshore marine environments are complex, but generalities are emerging and will strengthen with continued research.
